# Genetic Background-Dependent Expression of *Lr34*, *Lr67* and the *Lr46*-Associated Candidate Gene *Lr46-Glu2* in Wheat (*Triticum aestivum* L.) Highlights Contrasting Transcriptional Responses Following *Puccinia triticina* Infection

**DOI:** 10.3390/ijms27167391

**Published:** 2026-08-18

**Authors:** Julia Spychała, Aleksandra Noweiska, Roksana Bobrowska, Agnieszka Tomkowiak, Sylwia Mikołajczyk, Rafał Marcinkowski, Ada Dorczyk, Tadeusz Drzazga, Michał T. Kwiatek

**Affiliations:** 1 Plant Breeding and Acclimatization Institute, National Research Institute in Radzików, Radzików, 05-870 Błonie, Poland; j.spychala@ihar.edu.pl (J.S.); a.noweiska@ihar.edu.pl (A.N.); 2 Department of Genetics and Plant Breeding, Faculty of Agriculture, Horticulture and Biotechnology, Poznań University of Life Sciences, Dojazd 11, 60-632 Poznań, Poland; roksana.bobrowska@up.poznan.pl (R.B.); agnieszka.tomkowiak@up.poznan.pl (A.T.); sylwia.mikolajczyk@up.poznan.pl (S.M.); 3 Hodowla Roślin Strzelce Ltd., IHAR Group, Główna 20, 99-307 Strzelce, Poland; r_marcinkowski@hr-strzelce.pl; 4 Hodowla Roślin Smolice Ltd., IHAR Group, Smolice 146, 63-740 Kobylin, Poland; ada.dorczyk@hrsmolice.pl; 5 Małopolska Hodowla Roślin Ltd., Krajowa Grupa Spożywcza, Sportowa 21, 55-040 Kobierzyce, Poland; tdrzazga@mhr.com.pl

**Keywords:** adult plant resistance (APR), durable resistance, gene expression, genetic background, introgression, miRNA, *Puccinia triticina*, slow rusting resistance, wheat

## Abstract

Adult plant resistance (APR) is widely used in wheat breeding, but its behaviour across genetic backgrounds remains poorly understood. In this study, we analysed the expression of three APR loci (*Lr34*, *Lr46*, *Lr67*) following their introgression into elite winter wheat cultivars. BC_2_F_1_ populations derived from crosses between donor lines and commercial cultivars were evaluated under controlled infection with *Puccinia triticina* (*Pt*). Gene expression was assessed using RT-qPCR, and miRNA abundance was quantified by ddPCR at five time points (0–48 hours post-inoculation, hpi). Expression patterns differed markedly between genetic backgrounds, affecting both the magnitude and timing of gene activity. *Lr34* and *Lr67* showed the highest expression prior to inoculation, with no consistent or sustained induction following infection. In contrast, the candidate gene *Lr46-Glu2* displayed a clear tendency towards early induction, with peak expression typically observed at 6–12 hours post-inoculation, although the amplitude of this response varied among genotypes. Levels of miRNA varied across genotypes and time points and did not consistently reflect mRNA expression. The results suggest that the expression of APR-associated loci is influenced by genetic background. *Lr34* and *Lr67* showed predominantly baseline-associated expression, whereas the *Lr46-*associated candidate gene *Lr46-Glu2* displayed a more infection-responsive transcriptional profile following inoculation. These patterns are consistent with different transcriptional behaviours of the analysed genes but do not establish their functional contribution to resistance. Further phenotypic and functional validation will be required to determine the biological significance of these expression patterns.

## 1. Introduction

The sustainability of modern agriculture is increasingly challenged by the need to reduce chemical inputs while maintaining stable crop yields under rapidly changing environmental conditions. In wheat (*Triticum aestivum* L.), diseases caused by rust fungi remain a major constraint on production, and their management is further complicated by the emergence of new pathogen races and the shifting epidemiology associated with climate change [1,2]. In this context, host genetic resistance represents not only an effective but also an essential component of future crop protection strategies.

Plant resistance to pathogens is classically divided into race-specific (vertical) and race non-specific (horizontal) resistance. Race-specific resistance, typically conferred by major resistance (R) genes, relies on the recognition of pathogen effectors and often leads to a rapid hypersensitive response (HR). Although highly effective, such resistance is frequently overcome due to the evolutionary adaptability of pathogen populations [3,4]. By contrast, horizontal resistance, including adult plant resistance (APR), is characterized by partial, race non-specific effects that limit pathogen development rather than prevent infection. This form of resistance manifests through reduced infection frequency, prolonged latent period, and restricted pathogen growth, and is widely regarded as more durable under field conditions [5,6]. Importantly, APR reflects not a single defensive event but a coordinated modulation of host responses over time [7,8]. Among the loci associated with APR in wheat, *Lr34*, *Lr46* and *Lr67* have emerged as key genetic determinants of durable resistance. These loci confer partial resistance to multiple pathogens, including *Puccinia triticina*, *P. striiformis*, and *Blumeria graminis*, and are widely deployed in breeding programs [9,10,11]. Despite their similar phenotypic effects, their underlying molecular mechanisms appear to be fundamentally different.

The *Lr34* gene encodes an ATP-binding cassette (ABC) transporter of the ABCG family and is associated with constitutive activation of defence responses, including enhanced lignification and reinforcement of the cell wall [12]. This leads to increased resistance through structural barriers that limit pathogen penetration and spread [9]. In contrast, *Lr67* encodes a modified hexose transporter (STP13) that acts as a dominant-negative variant, impairing sugar uptake into host cells and thereby restricting the availability of nutrients to biotrophic pathogens [10,13]. Together, these genes illustrate two distinct mechanistic strategies: structural reinforcement and metabolic restriction.

In comparison, the molecular basis of *Lr46* (*Yr29*/*Sr58*/*Pm39*) remains unresolved. Although the phenotypic effects of *Lr46* have been well documented, such as reduced infection efficiency, extended latent period, and increased frequency of early aborted infection structures without host cell necrosis [14,15,16], the causal gene has not yet been identified. Mapping studies have localized *Lr46* to the distal region of chromosome 1BL, a region enriched in genes associated with defence and signalling [17].

Recent works provide new insights into the complexity of this locus. Expression analyses of candidate genes within the *Lr46* region revealed coordinated responses to pathogen infection, particularly among genes encoding glucan endo-1,3-β-glucosidases, receptor-like kinases, and transcriptional regulators. Notably, the gene *TraesCS1B02G454200.1*, a candidate gene for *Lr46-Glu2*, showed infection-induced expression consistent with a role in defence, while additional layers of regulation involving microRNAs, such as tae-miR5384-3p and tae-miR164, suggest a finely tuned regulatory network [17,18,19,20]. This type of multilayered regulation is increasingly recognized as a common feature of plant immunity, where small RNAs play a key role in fine-tuning defence responses and maintaining homeostasis between growth and stress signalling [21,22]. These findings challenge the classical view of resistance genes as single functional units and instead support a model in which *Lr46* may function as a complex genetic module, potentially corresponding to a tightly linked genomic region in which multiple genes and regulatory elements collectively contribute to the resistance phenotype. Hence, *Lr46* may function through a modulated basal immunity response, integrating pathogen perception, signalling, and controlled activation of defence mechanisms. Such a system would be consistent with the slow rusting phenotype, where pathogen growth is restricted rather than completely suppressed.

From a biological perspective, this raises an important question: to what extent do APR loci act independently, and to what extent do they interact with other resistance components within different genetic backgrounds? From a breeding perspective, the answer is equally critical. Durable resistance is most effectively achieved through the combination of vertical and horizontal resistance mechanisms, yet the stability and expression of APR genes such as *Lr34*, *Lr46* and *Lr67* may vary depending on the genetic context into which they are introduced. Although previous studies have characterised the expression of individual APR-associated genes and candidate genes within the *Lr46* region, much less is known about how these loci behave after introgression into different elite wheat backgrounds. In particular, the extent to which genetic background influences the temporal expression of *Lr34*, *Lr46*-associated genes and *Lr67* remains poorly understood. Addressing this gap is important for understanding the stability of APR-associated responses and their potential utilization in breeding programs. Therefore, the aim of the present study was to investigate the expression of *Lr34*, the candidate gene *Lr46-Glu2* and *Lr67* across different genetic backgrounds. These loci were introgressed into elite wheat cultivars currently used in agricultural practice, including Symetria, Euforia, and Medalistka. The study addresses whether the functional effects of APR genes are consistent across diverse genetic contexts and evaluates their potential for stable deployment in modern wheat breeding.

## 2. Results

### 2.1. Expression Analysis of APR-Associated Genes

**Figure 1:**
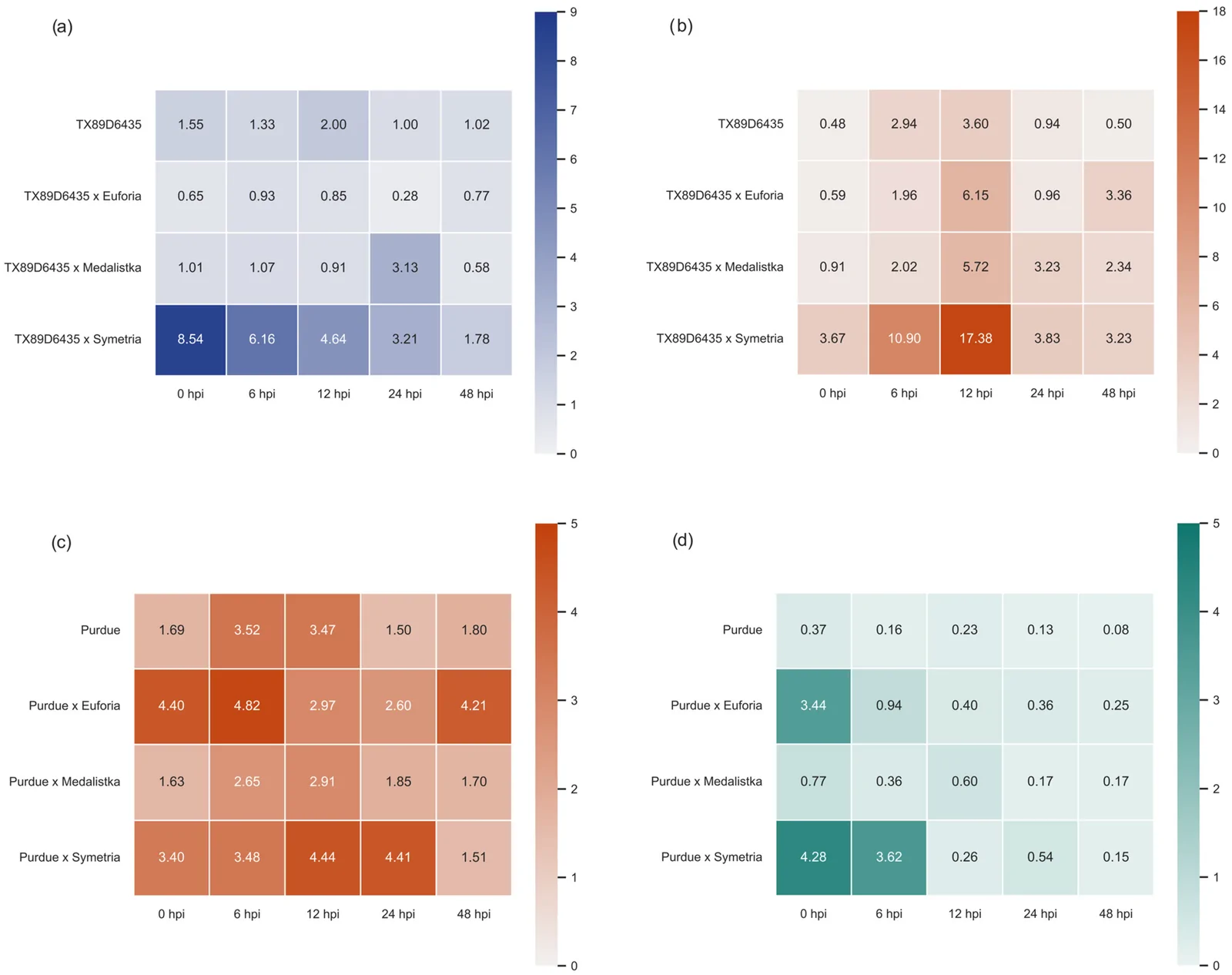
Heatmaps showing relative expression of (**a**) *Lr34*, (**b**,**c**) the *Lr46*-associated candidate gene *Lr46-Glu2*, and (**d**) *Lr67* in BC_2_F_1_ winter wheat genotypes at 0, 6, 12, 24 and 48 h after *P. triticina* inoculation. Values represent means from three biological replicates. Colour intensity indicates relative transcript abundance within each panel. Standard deviations and statistical comparisons are provided in Supplementary Tables S1–S3.

Expression of *Lr34* was analysed in three BC_2_F_1_ hybrid lines in which the *csLV34* marker was confirmed (Table 1). Expression varied markedly between the donor line TX89D6435 (TX) and BC_2_F_1_ hybrids and showed distinct temporal profiles following inoculation (Table S1; Figure 1a). In the donor genotype (TX), expression remained relatively stable across time points, with a moderate increase at 12 hpi (1.99) compared with the baseline at 0 hpi (1.54), followed by a return to near-initial levels at 24 and 48 hpi (1.00 and 1.02, respectively). In contrast, all BC_2_F_1_ hybrids exhibited altered expression patterns. The TX × Euforia combination showed consistently low expression across all time points, with only a slight increase at 6 hpi (0.93) relative to 0 hpi (0.65), followed by a decrease at 24 hpi (0.28) and partial recovery at 48 hpi (0.70). The TX × Medalistka hybrid displayed a distinct profile, with expression remaining relatively stable between 0 and 12 hpi (1.00–1.05), followed by a pronounced increase at 24 hpi (3.13), representing the highest value observed for this genotype, and a subsequent decline at 48 hpi (0.58). The increase observed at 24 hpi was statistically significant compared with the baseline level at 0 hpi (*p* = 0.0069). In the TX × Symetria hybrid, the highest overall expression among all analysed genotypes was observed at 0 hpi (8.54). Expression then decreased progressively over time, reaching 6.16 at 6 hpi, 4.64 at 12 hpi, 3.21 at 24 hpi, and 1.77 at 48 hpi. Overall, none of the BC_2_F_1_ hybrids reproduced the expression profile observed in the donor line TX. Instead, each hybrid combination exhibited a distinct temporal pattern, indicating a genotype-specific expression pattern on *Lr34* expression dynamics (Figure 1a).

Expression of the candidate gene *Lr46-Glu2* was assessed in all six BC_2_F_1_ hybrids (TX89D6435 × Euforia, Purdue × Euforia, Purdue × Medalistka, TX89D6435 × Medalistka, TX89D6435 × Symetria, Purdue × Symetria), consistent with the presence of the *csLV46G22* marker in all genotypes (Table 1). In the donor line TX, *Lr46-Glu2* expression increased markedly following inoculation, rising from 0.46 at 0 hpi to 2.94 at 6 hpi and reaching a peak at 12 hpi (3.59), followed by a decline at later time points (0.93 at 24 hpi and 0.50 at 48 hpi). Expression increases at 12 hpi were statistically significant relative to 0 hpi (*p* = 0.0149) (Table S2; Figure 1b,c). A comparable temporal pattern was observed in several TX-derived BC_2_F_1_ hybrids, although with substantial differences in expression magnitude. Among these, the TX × Symetria hybrid showed the highest overall expression levels across all analysed genotypes, with a strong increase from 3.67 at 0 hpi to 10.9 at 6 hpi and a pronounced peak at 12 hpi (17.38), followed by a sharp decrease at 24 hpi (3.82) and 48 hpi (3.23). Expression at 12 hpi was significantly higher than at 0 hpi (*p* = 0.0297). A similar, though less pronounced, pattern was observed in TX × Euforia and TX × Medalistka hybrids, both of which showed peak expression at 12 hpi (6.14 and 5.72, respectively). In TX × Euforia, expression at both 12 and 48 hpi was significantly higher than at 0 hpi (*p* < 0.01). In TX × Medalistka, elevated expression was maintained at 24 hpi (3.22), whereas in TX × Euforia expression declined at 24 hpi (0.96) but increased again at 48 hpi (3.36). In contrast, the donor line Purdue exhibited a more moderate and less dynamic expression profile, with relatively stable levels between 6 and 12 hpi (3.51 and 3.46, respectively) and no pronounced peak. This pattern was only partially reflected in Purdue-derived BC_2_F_1_ hybrids. The Purdue × Euforia hybrid showed relatively high expression across all time points, with values remaining above 2.6 and reaching 4.81 at 6 hpi and 4.21 at 48 hpi. The Purdue × Medalistka hybrid displayed a low-amplitude profile, with modest variation between 1.63 and 2.91. In the Purdue × Symetria hybrid, expression remained elevated between 0 and 24 hpi (3.39–4.44), with a slight peak at 12–24 hpi, followed by a decrease at 48 hpi (1.51). Overall, *Lr46-Glu2* expression showed a clear tendency towards early induction (6–12 hpi), particularly in TX-derived genotypes, although both the magnitude and temporal dynamics of expression varied substantially depending on genetic background (Figure 1b,c).

Expression of *Lr67* was analysed in the donor line Purdue and in three BC_2_F_1_ hybrid combinations carrying the target locus (Table S3; Figure 1d). In the donor genotype Purdue, expression was highest prior to inoculation (0 hpi; 0.36) and remained low at all subsequent time points, with a slight decrease observed at 6 hpi (0.16), followed by minor fluctuations between 0.08 and 0.23. In the BC_2_F_1_ hybrids, expression levels varied substantially depending on genetic background, but in most cases the highest values were observed at 0 hpi. The Purdue × Euforia hybrid showed high initial expression (3.44), followed by a sharp decrease at 6 hpi (0.93) and further decline at later time points (0.39–0.20). A similar trend was observed in the Purdue × Symetria hybrid, where expression was highest at 0 hpi (4.27) and remained relatively high at 6 hpi (3.62), followed by a pronounced decrease at 12 hpi (0.25) and low levels thereafter. Expression at 12 and 48 hpi was significantly lower than at 0 hpi (*p* < 0.05). In contrast, the Purdue × Medalistka hybrid exhibited generally low expression across all time points, with only a modest peak at 0 hpi (0.76) and no clear temporal trend following inoculation. Overall, *Lr67* expression was highest prior to inoculation and did not show consistent induction in response to infection. Instead, expression levels tended to decrease or remain low at later time points, with substantial variation in magnitude between genotypes (Figure 1d).

### 2.2. Expression Analysis of miRNAs Associated with Resistance Genes

**Figure 2:**
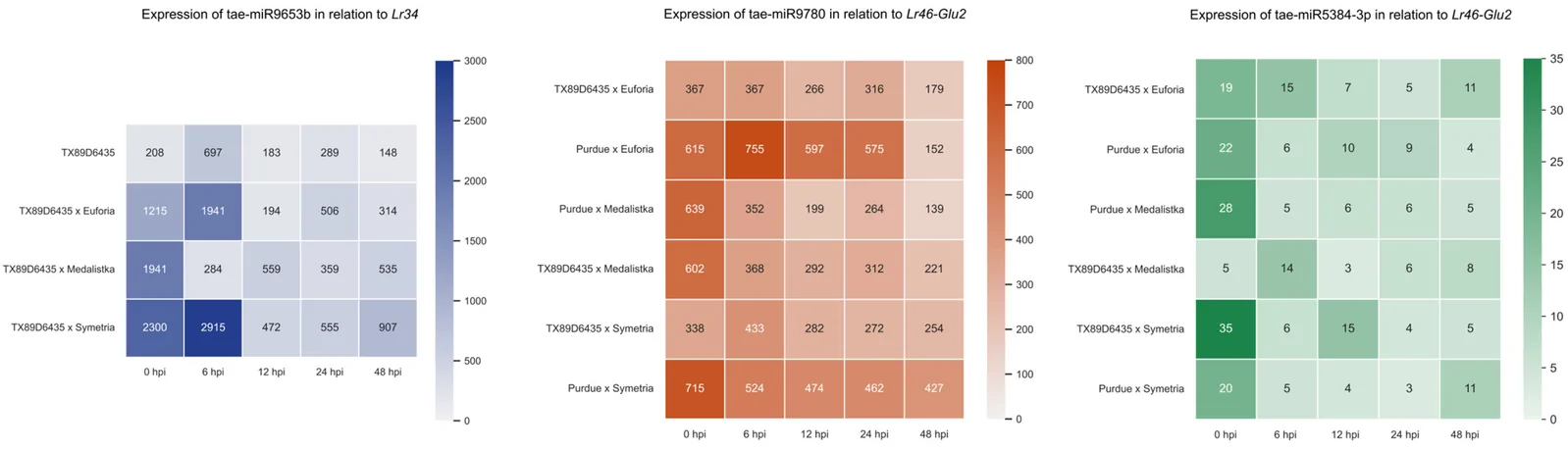
Expression profiles of miRNAs associated with *Lr34* and *Lr46* in leaf tissues following infection with *Puccinia triticina* (*Pt*). miRNA expression was quantified by droplet digital PCR (ddPCR) in BC_2_F_1_ winter wheat hybrids and donor lines at 0, 6, 12, 24, and 48 hours post-inoculation (hpi).

The expression of miRNAs associated with adult plant resistance loci showed genotype- and time-dependent variation (Tables S4–S6; Figure 2). For tae-miR9653b, associated with *Lr34*, the donor line TX89D6435 exhibited a clear peak in expression at 6 h post-inoculation (hpi) (Supplementary Table S4; Figure 2). A comparable expression pattern was observed only in the hybrid combination TX89D6435 × Euforia. In contrast, the remaining hybrid genotypes showed the highest accumulation of this miRNA at 0 hpi, indicating a high basal level prior to inoculation. Notably, the highest overall copy number of tae-miR9653b was detected at 6 hpi in the TX89D6435 × Symetria hybrid (Table S4; Figure 2).

Expression of tae-miR5384-3p, associated with *Lr46–Glu2*, displayed substantial variability across genotypes and time points (Table S5; Figure 2). The highest expression level was recorded at 6 hpi in the Purdue × Euforia hybrid (755 copies μL^−1^). However, in most of the remaining hybrids, expression was highest at 0 hpi and was not exceeded at later time points following inoculation. To examine whether tae-miR5384-3p abundance was associated with the expression of the *Lr46*-associated candidate gene *Lr46-Glu2*, Pearson correlation analysis was performed using paired measurements from six BC_2_F_1_ genotypes across five sampling time points. Across the complete dataset (*n* = 30), no significant correlation was detected (r = −0.070, *p* = 0.713; Table S7). Separate exploratory analyses of TX-derived and Purdue-derived genotypes likewise revealed no significant associations (r = −0.132, *p* = 0.640 and r = 0.310, *p* = 0.261, respectively). Genotype-specific correlations were also non-significant (Table S7), indicating that tae-miR5384-3p abundance did not show a consistent linear relationship with *Lr46-Glu2* transcript levels under the conditions analysed.

In the case of tae-miR8780, also associated with *Lr46–Glu2*, overall expression levels were low across all analysed genotypes (Table S6; Figure 2). This pattern was consistent with observations from previous experimental seasons. ddPCR analysis indicated that, similarly to other miRNAs, the highest expression was generally observed at 0 hpi. An exception was the TX89D6435 × Medalistka hybrid, where peak expression occurred at 6 hpi (Table S6; Figure 2).

## 3. Discussion

The present study provides new insight into the functional behaviour of *Lr34*, *Lr46* and *Lr67* across contrasting genetic backgrounds. By integrating expression data from donor lines with BC_2_F_1_ hybrids, it was possible to capture not only static expression profiles, but also the way in which APR loci are accommodated within elite wheat germplasm. Because the primary objective of this study was to analyse gene expression dynamics following inoculation, the results provide insight into the regulation of APR-associated loci rather than direct evidence of resistance levels in the analysed genotypes. The use of BC_2_F_1_ material should also be considered when interpreting the results, as segregation of genomic regions outside the marker-confirmed APR loci may have contributed to the observed variation in gene expression among genotypes.

### 3.1. Genetic Background as a Primary Determinant of APR Expression

The analysed APR loci displayed substantial variation in expression among genetic backgrounds, highlighting the importance of the recipient genomic context. This is consistent with previous reports demonstrating that gene expression in complex traits depends on epistatic interactions and regulatory context [23,24]. None of the analysed BC_2_F_1_ hybrids reproduced the expression profiles observed in the donor lines. Instead, each genetic background imposed a distinct temporal pattern, affecting both the magnitude and timing of gene expression. This effect was particularly evident for *Lr46-Glu2*, where TX-derived genotypes showed a clear tendency towards early induction (6–12 hpi), whereas Purdue-derived combinations exhibited more moderate and less dynamic profiles. Similarly, *Lr67* expression differed substantially in magnitude between genotypes, although the general tendency towards higher baseline expression followed by a decline after inoculation was preserved. These observations indicate that introgressed loci do not retain a fixed expression pattern but are reconfigured within the regulatory environment of the recipient genome. This process likely reflects interactions between cis-regulatory elements and trans-acting factors originating from different parental backgrounds, leading to non-additive expression patterns consistent with allele-specific regulation and epigenetic modulation [25,26]. At the BC_2_F_1_ stage, expression profiles showed consistent early induction of *Lr46-Glu2* and baseline-dominated expression of *Lr34* and *Lr67*, suggesting that some regulatory features become stabilized during introgression, although expression profiles remain influenced by the recipient genome and do not fully converge towards those observed in the donor lines.

### 3.2. Contrasting Temporal Expression Patterns of Lr34, Lr67 and the Lr46-Associated Candidate Gene Lr46-Glu2

Beyond background effects, the analysed genes exhibited clearly different temporal expression patterns following inoculation. Both *Lr34* and *Lr67* showed the highest expression predominantly prior to inoculation (0 hpi), with no consistent or sustained induction at later time points. In the case of *Lr34*, expression remained relatively stable, with moderate fluctuations over time. An additional observation was the exceptionally high baseline expression of *Lr34* in some genetic backgrounds, particularly in the TX × Symetria combination. This may indicate differences in constitutive regulation among recipient genomes and suggests that the transcriptional activity of APR-associated loci can be substantially modified following introgression. However, the biological significance of this pattern requires further investigation. For *Lr67*, expression tended to decrease after inoculation, particularly in genotypes with high initial expression, although its temporal dynamics remained variable depending on genetic background. These patterns are not consistent with a classical inducible transcriptional response and instead indicate predominantly baseline-associated expression under the conditions analysed. The known molecular functions of *Lr34* and *Lr67* provide biological context for the observed transcriptional profiles. *Lr34* encodes an ABC transporter associated with defence-related processes, including cell wall reinforcement [9,12], whereas *Lr67* encodes a modified hexose transporter affecting carbohydrate partitioning [10,13]. However, the present study assessed transcript abundance only and therefore does not demonstrate that the observed expression patterns correspond directly to constitutive defence activity.

In contrast, the *Lr46*-associated candidate gene *Lr46-Glu2* displayed a different temporal expression profile. In TX-derived genotypes, transcript levels increased after inoculation and generally peaked at 12 hpi before declining at later time points. This profile is consistent with an early transcriptional response to inoculation but does not establish a functional role for *Lr46*-*Glu2* in pathogen signalling or resistance. The magnitude of this response varied substantially among genetic backgrounds, with TX × Symetria showing the strongest increase. These observations indicate that the transcriptional behaviour of *Lr46-Glu2* is influenced by genetic background. Compared with *Lr34* and *Lr67*, the candidate gene *Lr46-Glu2* displayed a more responsive expression profile following inoculation. Although the molecular identity of the *Lr46* resistance gene remains unresolved, this pattern may reflect regulatory processes associated with the *Lr46* genomic region. Particularly noteworthy was the TX × Symetria genotype, which exhibited the strongest induction of *Lr46-Glu2* among all analysed combinations. This observation suggests that the Symetria background may contain factors enhancing the transcriptional response of genes located within the *Lr46* genomic region. Although the mechanisms underlying this effect remain unknown, it further illustrates the strong influence of genotype on APR-associated expression patterns. This interpretation is supported by previous studies indicating that the *Lr46* locus is associated with coordinated expression of defence-related genes and miRNA-mediated regulation [18,20].

### 3.3. Consistency of APR Expression Patterns Across Developmental Systems

An important question arising from this study is whether the observed expression patterns are specific to the analysed winter wheat material, or whether they reflect more general properties of APR loci. To address this, the present results are compared below with independent expression analyses performed in hybrid populations of spring wheat under comparable experimental conditions [19]. Despite differences in genetic background, developmental type and generation structure, several similarities were observed between the two studies. *Lr34* consistently showed low and relatively stable expression, with no evidence of strong induction following inoculation. Similarly, *Lr46-Glu2* exhibited a clear tendency towards early induction, with peak expression observed at 6–12 hpi in both winter and spring materials. This temporal pattern was reproducible across different genetic combinations, although the magnitude of induction varied between genotypes. In contrast, *Lr67* displayed the highest variability across systems. While a baseline-dominated expression pattern was frequently observed, particularly in genotypes with high initial expression, the temporal dynamics of this gene were less consistent and more strongly influenced by genetic background. In spring wheat, more complex expression profiles were observed in some combinations, including transient increases at later time points, further indicating that *Lr67* is particularly sensitive to context-dependent regulatory effects [19]. Taken together, these observations indicate that the fundamental modes of *Lr34*, *Lr46* and *Lr67* gene expression are consistent with our previous findings obtained in spring wheat. However, the amplitude and precise timing of these responses remain strongly dependent on genotype and developmental context. Importantly, the combination of results obtained in spring and winter wheat provides a coherent and complementary view of APR gene behaviour across contrasting developmental systems. While previous analyses focused on spring wheat hybrid populations [19], the present study extends these observations to winter wheat and more advanced introgression stages. Together, these datasets suggest that similar regulatory trends may occur in both spring and winter wheat. However, the magnitude and timing of these responses remain dependent on genetic background, developmental context and introgression stage, and additional studies will be required to determine their broader biological significance.

### 3.4. Regulatory Complexity and the Role of miRNAs

The analysed miRNAs showed genotype- and time-dependent variation but did not consistently mirror the expression of their putative target genes. Small RNAs are widely recognised as components of post-transcriptional regulatory networks and may contribute to the modulation of plant responses to biotic stress [21,22]. However, the present data do not provide direct evidence for such regulatory interactions. To examine whether tae-miR5384-3p abundance was associated with expression of the *Lr46-*associated candidate gene *Lr46-Glu2*, Pearson correlation analysis was performed using paired measurements from six BC_2_F_1_ genotypes across five sampling time points. Across the complete dataset (*n* = 30), no significant correlation was detected (Pearson’s r = −0.070, *p* = 0.713; Table S7). Separate exploratory analyses of TX-derived and Purdue-derived genotypes likewise revealed no significant associations, and none of the genotype-specific correlations reached statistical significance (Table S7). Although individual genotypes differed in the direction and magnitude of the correlation coefficients, the small number of temporal observations within each genotype limits interpretation of these exploratory analyses. Overall, the data provide no evidence for a consistent linear relationship between tae-miR5384-3p abundance and *Lr46-Glu2* transcript accumulation under the conditions analysed. Therefore, a causal miRNA–target relationship cannot be inferred from the present expression data and would require direct functional validation.

### 3.5. Study Limitations

The present study was designed to characterise transcriptional responses of APR-associated genes following *P. triticina* inoculation and has several limitations. Resistance phenotypes, including quantitative disease severity, were not evaluated, and pathogen biomass was not quantified. Although the appearance of typical *P. triticina* symptoms confirmed successful infection, these observations were not used as a quantitative measure of resistance. Protein accumulation was not assessed, and the proposed relationships between miRNAs and their putative target genes were not functionally validated. Consequently, the regulatory mechanisms discussed in this study are inferred from temporal expression patterns and should not be interpreted as evidence of causal interactions or functional contributions to resistance. In addition, the BC_2_F_1_ material remains segregating for genomic regions outside the marker-confirmed APR loci, which may have contributed to variation among genotypes. Further validation in more advanced generations and additional genetic backgrounds, together with phenotypic, pathogen-quantification and protein-level analyses, will therefore be required to establish the biological significance of the observed transcriptional patterns.

## 4. Materials and Methods

### 4.1. Plant Material

Plant material consisted of six BC_2_F_1_ wheat hybrids derived from backcrossing donor lines, TX89D6435 (PI 584759) and Purdue (Cltr 13227) with breeding genotypes originating from commercial partners (Table 1). Donor lines were provided by the National Small Grains Germplasm Facility, National Small Grains Collection in Aberdeen, ID, USA. Winter wheat BC_2_F_1_ hybrid seeds were provided by three breeding companies: Hodowla Roślin Smolice Ltd., (cv. Symetria), Małopolska Hodowla Roślin Ltd., (cv. Medalistka), and Hodowla Roślin Strzelce Ltd., (cv. Euforia). The experiment comprised eight genotypes (six BC_2_F_1_ hybrids and two donor cultivars), grown in three biological replicates. Leaf tissue was collected before inoculation (0 hpi) and at 6, 12, 24 and 48 hpi (hours post-inoculation) following inoculation. Samples collected at 0 hpi were used as the pre-inoculation baseline for each genotype.

### 4.2. Molecular Marker Analysis

Genomic DNA was extracted from leaf tissue of all hybrid genotypes and parental winter wheat lines. Samples were collected at the two-leaf stage (BBCH 12), immediately frozen, and stored at −80 °C until further processing. DNA isolation was performed using the GeneMATRIX Plant & Fungi DNA Purification Kit (EURx Ltd., Gdańsk, Poland), following the manufacturer’s protocol. DNA quality and concentration were suitable for downstream PCR analyses. DNA concentration and purity were assessed spectrophotometrically using a NanoDrop Ultra Microvolume UV-Vis (Thermo Fischer Scientific, Waltham, MA, USA). DNA quality was evaluated based on A260/A280 absorbance ratios, and only samples showing appropriate purity and concentration were used for downstream PCR analyses.

Molecular screening focused on the identification of key adult plant resistance (APR) loci using gene-linked markers. The following markers were applied: *csLV34* for *Lr34* [27]), *csLV46G22* for *Lr46* (unpublished marker developed byProf. E. Lagudah’s research team), and *Xcfd71* together with *Xcfd23* for *Lr67* [28].. PCR reactions were carried out using a standard HotStart ReadyMix (NIPPON Genetics Europe GmbH, Düren, Germany), under optimized conditions, with each sample amplified at least twice to ensure reproducibility. For the CAPS marker *csLV46G22*, PCR products were digested with the restriction enzyme *BspEI* (Thermo Fisher Scientific, Waltham, MA, USA) prior to analysis. Amplified fragments were separated on 2% agarose gels and visualized under UV light. Marker profiles were used to classify genotypes according to the presence or absence of the target resistance loci. This approach enabled efficient marker-assisted selection of lines carrying the analysed APR genes.

### 4.3. Experimental Setup of BC_2_F_1_hybrids Under Controlled Conditions

The experiment with BC_2_F_1_ hybrids was conducted under controlled environmental conditions in a phytotron facility at the Poznań Centre for Advanced Technologies (PCAT), Adam Mickiewicz University, Poznań, Poland. Plants were grown in growth chambers equipped with systems enabling precise control of temperature, humidity, and light conditions.

BC_2_F_1_ plants were cultivated under controlled conditions until the two-leaf stage (BBCH 12). During this initial phase, environmental parameters were maintained as follows: daytime temperature of 18–20 °C, nighttime temperature of 14–16 °C, a 14 h photoperiod, relative humidity of 60–70%, and a light intensity of 250–300 μmol m^−2^ s^−1^. After reaching the appropriate developmental stage, plants were subjected to vernalization for 8 weeks. During this period, growth chamber conditions were adjusted to a temperature of 4–6 °C, a 10 h photoperiod, relative humidity of 60–70%, and a light intensity of 150–250 μmol m^−2^ s^−1^. All genotypes were subjected to the same vernalisation regime and reached the adult plant stage before inoculation.

Following vernalization, plants were transferred to conditions promoting further growth and development to the adult stage. The environmental parameters were set to 20–22 °C during the day and 16–18 °C at night, with a 16 h photoperiod, relative humidity of 70–80%, and a light intensity of 300–350 μmol m^−2^ s^−1^. At the adult plant stage, biotic stress was induced by inoculation with urediniospores solution of *P. triticina* collected from naturally infected wheat fields during the experimental season. A mixed inoculum was used because the analysed APR loci are associated with race non-specific resistance and the objective was to evaluate gene expression responses under a broad infection stimulus representative of field conditions. BC_2_F_1_ wheat hybrids were inoculated by spraying whole plants with a suspension of urediniospores at a concentration of approximately 5 × 10^5^ spores mL^−1^ in water supplemented with Tween 20 (Merck-Sigma Aldrich, Burlington, MA, USA). Successful infection was verified by the appearance of typical *P. triticina* infection symptoms on inoculated plants.

### 4.4. RNA and miRNA Isolation, cDNA Synthesis and Expression Analysis

Leaf tissue was collected from six BC_2_F_1_ hybrids and the donor lines, with three biological replicates per genotype and two treatments (inoculated and non-inoculated). Samples were taken before inoculation (0 h post-inoculation; hpi) and at 6, 12, 24 and 48 hpi. In total, 240 samples were obtained (8 genotypes × 3 biological replicates × 5 time points × 2 treatments). Collected leaves were immediately frozen in liquid nitrogen and stored at −80 °C until processing. Total RNA was extracted using the SV Total RNA Isolation System (Promega, Madison, WI, USA) according to the manufacturer’s instructions. RNA concentration and quality were assessed spectrophotometrically (Thermo Fischer Scientific, USA) in A260/A280 absorbance ratio. For mRNA analyses, first-strand cDNA was synthesised from 1 µg of total RNA using the iScript cDNA Synthesis Kit (Bio-Rad, Hercules, CA, USA), following thermal conditions with modifications (5 min at 25 °C, 60 min at 46 °C, 1 min at 95 °C). For miRNA analysis, cDNA synthesis was performed using a stem–loop RT approach with SuperScript™ IV Reverse Transcriptase (Thermo Fisher Scientific, Waltham, MA, USA). Gene-specific primers used for RT-qPCR analysis of *Lr34, Lr67* and the candidate gene *Lr46-Glu2* were described in our previous studies [20,29]. For miRNA expression analyses, stem-loop RT primers were designed based on the sequences of miRNAs predicted to target *Lr34* and the candidate gene *Lr46-Glu2*, and their sequences were also reported previously [20,29].

Gene expression was analysed using two complementary approaches. Quantitative real-time PCR (RT-qPCR) was performed with iTaq Universal SYBR Green Supermix (Bio-Rad, Hercules, CA, USA) on a CFX Connect Real-Time PCR Detection System (Bio-Rad, Hercules, CA, USA). Primers for miRNA reverse transcription and ddPCR were designed using the Integrated DNA Technologies website, in accordance with the protocols [30,31] (Forero et al., 2019; Kramer, 2011). Primers targeted *Lr34*, *Lr67*, and the candidate gene for *Lr46* (*Glu2*) were designed and their sequences were described in the research team’s previous work [20,29]. In parallel, droplet digital PCR (ddPCR) was used for absolute quantification of miRNA-derived cDNA. Reactions were prepared according to the manufacturer’s guidelines, and droplet generation enabled partitioning of individual templates into independent PCR reactions. The ddPCR products were quantitatively analysed in a QX100 Droplet Reader (Bio-Rad, Inc., Hercules, CA, USA).

### 4.5. Statistical Analyses

Data were analysed based on positive and negative droplet discrimination using Poisson statistics. Gene expression levels of *Lr34*, *Lr46-Glu2* (candidate), and *Lr67* were determined from three biological replicates for each genotype and time point. Mean values obtained from biological replicates were used for subsequent statistical analyses and are presented in Tables S1–S3. Two reference genes (*TUBβ* and *ARF*) were included for normalisation. Relative gene expression was calculated using the 2^−ΔΔCt^ method. Expression values were normalized against two reference genes (*TUBβ* and *ARF*) selected for validation in a previous study by our research team [29]. Samples collected before inoculation (0 hpi) were used as calibrators. Statistical comparisons were performed between individual time points and the corresponding 0 hpi control within each genotype. Homogeneity of variance was assessed using Levene’s test (Brown–Forsythe variant), and differences between means were evaluated using Student’s *t*-test. The Kolmogorov–Smirnov test (K–S) was used to assess data distribution [32]. The analysis focused on temporal changes in expression within individual genotypes; genotype-by-time interactions were not formally evaluated. Paired measurements of tae-miR5384-3p abundance determined by ddPCR and mean relative expression of the *Lr46-*associated candidate gene *Lr46-Glu2* determined by RT-qPCR at 0, 6, 12, 24 and 48 hours post-inoculation (hpi). The overall Pearson correlation was calculated using 30 paired genotype × time-point observations. Additional correlations calculated separately for donor-background groups and individual genotypes are presented as exploratory analyses. Statistical significance was assessed at *p* < 0.05. No significant correlation was detected in the complete dataset or in any of the analysed subsets (Table S7).

## 5. Conclusions

The present study shows that APR-associated genes exhibit distinct temporal expression patterns shaped by genetic background, with *Lr34* and *Lr67* showing predominantly baseline-associated expression and the *Lr46*-associated candidate gene *Lr46-Glu2* showing a more infection-responsive profile. The results indicate that the observed expression patterns of the *Lr34*, *Lr46-Glu2*, and *Lr67* genes are not specific solely to the winter wheat material analysed. A comparison of the obtained results with those presented in a series of publications by our team from 2023–2025 on spring wheat revealed similar overall expression profiles. *Lr34* was characterized by stable, relatively low expression and a lack of clear induction following inoculation, whereas *Lr46-Glu2* exhibited a consistent tendency toward early induction, reaching its highest expression level at 6–12 hpi. The greatest variability was observed for *Lr67*, whose expression was more strongly dependent on the genetic background. It can therefore be concluded that the basic expression patterns of the analysed genes are relatively consistent across different wheat materials; however, their intensity and temporal dynamics depend on the genotype and developmental context. The biological significance of the observed changes requires further validation through phenotypic and functional studies, including, among other things, pathogen quantification and protein-level analyses. These observations describe contrasting transcriptional responses and should not be interpreted as evidence that the analysed genes perform distinct functional roles in resistance.

## Figures and Tables

**Table 1:** Marker-based detection of resistance genes (*Lr34*, *Lr46*, *Lr67*) in parental and BC_2_F_1_ materials.

**Plant Material**	***Lr34***	***Lr46***	***Lr67***
Donors			
TX89D6435	+	+	-
Purdue	-	+	+
Acceptors			
Euforia	-	-	-
Medalistka	-	-	-
Symetria	-	-	-
BC_2_F_1_ hybrids			
TX89D6435 × Euforia	+	+	-
TX89D6435 × Medalistka	+	+	-
TX89D6435 × Symetria	+	+	-
Purdue × Euforia	-	+	+
Purdue × Medalistka	-	+	+
Purdue × Symetria	-	+	+

## Data Availability

The datasets generated and analysed during the current study are stored on institutional servers and are available from the corresponding author upon reasonable request. Data are not deposited in a public repository due to their size and institutional data management policies; however, all data necessary to support the conclusions of this study are available upon request.

## Supplementary Materials

The following supporting information can be downloaded at: https://www.mdpi.com/article/10.3390/ijms27167391/s1.
