# Supplementary material for: Genetic Background-Dependent Expression of Lr34, Lr67 and the Lr46-Associated Candidate Gene Lr46-Glu2 in Wheat (Triticum aestivum L.) Highlights Contrasting Transcriptional Responses Following Puccinia triticina Infection
Source: Int J Mol Sci. 2026 Aug 18;27(16):7391. doi: 10.3390/ijms27167391 (PMC13513563; doi:10.3390/ijms27167391)
Supplement: Supplementary file 1 [file ijms-27-07391-s001.zip › Table S1.pdf]

Table. S1. Statistical analysis of *Lr34* expression in BC_2F_1 hybrid lines and the donor line TX89D6435 (TX). Statistically significant differences ($p < 0.05$) are indicated in green.

**Plant material**	**hai***	**Mean**	**Standard deviation**	**T/0h**	**K-S**	**Levene's**	**t-Student**
TX × Euforia	00h	0.654163333	0.278626411		0.53548		
TX × Euforia	06h	0.930676667	0.108859872	1.422698		0.3237	0.261206
TX × Euforia	12h	0.849976667	0.123616143	1.299334		0.3689	0.415008
TX × Euforia	24h	0.281356667	0.039379354	0.430102		0.166	0.134268
TX × Euforia	48h	0.77269	0.46381423	1.181188		0.5719	0.772183
TX × Medalistka	00h	1.007776667	0.422475646		0.10556		
TX × Medalistka	06h	1.06755	0.112882596	1.059312		0.2346	0.856138
TX × Medalistka	12h	0.914166667	0.104526528	0.907112		0.222	0.776157
TX × Medalistka	24h	3.132426667	0.40883901	3.108255		0.9447	0.00693
TX × Medalistka	48h	0.582196667	0.111354279	0.577704		0.2349	0.240394
TX × Symetria	00h	8.540406667	4.602922456		0.5418		
TX × Symetria	06h	6.16315	1.07845	0.721646		0.2463	0.512799
TX × Symetria	12h	4.643943333	1.178182814	0.543761		0.2854	0.310653
TX × Symetria	24h	3.211416667	0.928630495	0.376026		0.2522	0.183773
TX × Symetria	48h	1.777456667	0.705191739	0.208123		0.225	0.109206
TX	00h	1.54737	0.439605681		0.83373		
TX	06h	1.334323333	0.219503002	0.862317		0.4563	0.572907
TX	12h	1.99602	0.485302952	1.289944		0.9619	0.387431
TX	24h	1.002135992	0.129816529	0.647638		0.2858	0.16782
TX	48h	1.021476667	0.617673578	0.660137		0.668	0.382141
