# Supplementary material for: Genetic Background-Dependent Expression of Lr34, Lr67 and the Lr46-Associated Candidate Gene Lr46-Glu2 in Wheat (Triticum aestivum L.) Highlights Contrasting Transcriptional Responses Following Puccinia triticina Infection
Source: Int J Mol Sci. 2026 Aug 18;27(16):7391. doi: 10.3390/ijms27167391 (PMC13513563; doi:10.3390/ijms27167391)
Supplement: Supplementary file 1 [file ijms-27-07391-s001.zip › Table S2.pdf]

Table. S2. Statistical analysis of *Lr46–Glu2* expression in BC_2F_1 hybrid lines and the donor lines TX89D6435 (TX) and Purdue. Statistically significant differences ($p < 0.05$) are indicated in green.

Plant material	Time	Mean	Standard deviation	T/0h	K-S	Levene's	t-Student
TX × Euforia	00h	0.585863	0.309824		0.53891		
TX × Euforia	06h	1.95651	0.814387	3.339533		0.3667	0.090147
TX × Euforia	12h	6.148727	0.771943	10.49516		0.3427	0.000697
TX × Euforia	24h	0.963597	0.256683	1.644746		0.8289	0.254972
TX × Euforia	48h	3.363223	0.652606	5.740628		0.6219	0.005554
Purdue × Euforia	00h	4.395653	1.675216		0.98273		
Purdue × Euforia	06h	4.81612	0.587345	1.095655		0.5777	0.75448
Purdue × Euforia	12h	2.971887	0.606413	0.676097		0.5758	0.321581
Purdue × Euforia	24h	2.60181	0.643154	0.591905		0.6053	0.230327
Purdue × Euforia	48h	4.211077	0.578413	0.958009		0.5748	0.890031
Purdue × Medalistka	00h	1.63388	0.070447		0.03841		
Purdue × Medalistka	06h	2.65033	0.021278	1.622108		0.4433	0.000041
Purdue × Medalistka	12h	2.911443	1.161828	1.78192		0.2533	0.195557
Purdue × Medalistka	24h	1.854273	0.230584	1.13489		0.2809	0.265722
Purdue × Medalistka	48h	1.69557	4.425974	5.934077		0.1671	0.061613
TX × Medalistka	00h	0.913003	0.532834		0.37615		
TX × Medalistka	06h	2.02216	0.25349	2.214844		0.5213	0.056487
TX × Medalistka	12h	5.721967	2.740966	6.267191		0.4592	0.071546
TX × Medalistka	24h	3.227387	0.743981	3.534912		0.7207	0.023237
TX × Medalistka	48h	2.33575	0.741823	2.558315		0.6921	0.092349
TX × Symetria	00h	3.67479	1.195656		0.23681		
TX × Symetria	06h	10.90005	7.96146	2.966169		0.184	0.197013
TX × Symetria	12h	17.38316	5.739228	4.730383		0.2699	0.02974
TX × Symetria	24h	3.82968	2.105473	1.042149		0.579	0.932265
TX × Symetria	48h	3.23166	1.793443	0.879414		0.8089	0.785702
Purdue × Symetria	00h	3.39635	1.189573		0.44032		
Purdue × Symetria	06h	3.47521	0.742278	1.023219		0.7395	0.940425
Purdue × Symetria	12h	4.44406	2.033402	1.308481		0.5748	0.563511
Purdue × Symetria	24h	4.412093	1.636111	1.299069		0.7649	0.516835
Purdue × Symetria	48h	1.514223	0.664967	0.445838		0.6936	0.122519
TX	00h	0.475697	0.170281		0.28		
TX	06h	2.937583	1.294526	6.175329		0.1619	0.056008
TX	12h	3.59742	1.063406	7.562424		0.3184	0.014861
TX	24h	0.938694	0.43117	1.973304		0.4304	0.230681
TX	48h	0.502327	0.176942	1.055981		0.9348	0.88554
Purdue	00h	1.688897	0.890681		0.71035		
Purdue	06h	3.51591	0.899132	2.081779		0.9808	0.110749
Purdue	12h	3.469	1.063406	2.054004		0.9225	0.099839
Purdue	24h	1.49518	0.239193	0.8853		0.4894	0.781211
Purdue	48h	1.79732	0.885403	1.064198		0.9196	0.908714
