# Supplementary material for: Genetic Background-Dependent Expression of Lr34, Lr67 and the Lr46-Associated Candidate Gene Lr46-Glu2 in Wheat (Triticum aestivum L.) Highlights Contrasting Transcriptional Responses Following Puccinia triticina Infection
Source: Int J Mol Sci. 2026 Aug 18;27(16):7391. doi: 10.3390/ijms27167391 (PMC13513563; doi:10.3390/ijms27167391)
Supplement: Supplementary file 1 [file ijms-27-07391-s001.zip › Table S3.pdf]

Table. S3. Statistical analysis of *Lr67* expression in $BC_2F_1$ hybrid lines and the donor line Purdue. Statistically significant differences ($p < 0.05$) are indicated in green.

| Plant material | Time | Mean | Standard deviation | T/0h | K-S | Levene's | t-Student |
|---|---|---|---|---|---|---|---|
| Purdue × Euforia | 00h | 3.44195 | 3.521282977 | | 0.00539 | | |
| Purdue × Euforia | 06h | 0.937287 | 0.134743963 | 0.272313 | | 0.385 | 0.371681 |
| Purdue × Euforia | 12h | 0.399637 | 0.072590545 | 0.116108 | | 0.3785 | 0.288933 |
| Purdue × Euforia | 24h | 0.356233 | 0.127147675 | 0.103498 | | 0.3825 | 0.283255 |
| Purdue × Euforia | 48h | 0.2527 | 0.119001068 | 0.073418 | | 0.3823 | 0.269696 |
| Purdue × Medalistka | 00h | 0.768723 | 0.293815578 | | 0.72207 | | |
| Purdue × Medalistka | 06h | 0.357537 | 0.006592245 | 0.465104 | | 0.2193 | 0.118985 |
| Purdue × Medalistka | 12h | 0.604117 | 0.07061315 | 0.78587 | | 0.3302 | 0.484061 |
| Purdue × Medalistka | 24h | 0.17356 | 0.010463145 | 0.225777 | | 0.2247 | 0.045797 |
| Purdue × Medalistka | 48h | 0.165983 | 0.070685562 | 0.215921 | | 0.3329 | 0.047796 |
| Purdue × Symetria | 00h | 4.27745 | 1.988142904 | | 0.05889 | | |
| Purdue × Symetria | 06h | 3.622367 | 3.729142679 | 0.846852 | | 0.6577 | 0.837211 |
| Purdue × Symetria | 12h | 0.25787 | 0.027879842 | 0.060286 | | 0.3557 | 0.045979 |
| Purdue × Symetria | 24h | 0.539517 | 0.091844973 | 0.12613 | | 0.3697 | 0.056623 |
| Purdue × Symetria | 48h | 0.14726 | 0.020553584 | 0.034427 | | 0.3535 | 0.042484 |
| Purdue | 00h | 0.365117 | 0.136705928 | | 0.43634 | | |
| Purdue | 06h | 0.162207 | 0.013519177 | 0.44426 | | 0.2373 | 0.104961 |
| Purdue | 12h | 0.232947 | 0.059614393 | 0.638006 | | 0.4676 | 0.278354 |
| Purdue | 24h | 0.13124 | 0.026548274 | 0.359447 | | 0.287 | 0.076398 |
| Purdue | 48h | 0.079643 | 0.011394201 | 0.218131 | | 0.2278 | 0.042263 |
