# Supplementary material for: Genetic Background-Dependent Expression of Lr34, Lr67 and the Lr46-Associated Candidate Gene Lr46-Glu2 in Wheat (Triticum aestivum L.) Highlights Contrasting Transcriptional Responses Following Puccinia triticina Infection
Source: Int J Mol Sci. 2026 Aug 18;27(16):7391. doi: 10.3390/ijms27167391 (PMC13513563; doi:10.3390/ijms27167391)
Supplement: Supplementary file 1 [file ijms-27-07391-s001.zip › Table S5.pdf]

Table. S5. Expression levels of miR5384-3p in the analysed winter wheat genotypes before inoculation and at 6-, 12-, 24-, and 48-hours post-inoculation (hpi). Values represent means of biological replicates (including technical replicates).

**Plant material**	**miRNA copy number/µL at:**				
	0 hpi	*6 hpi*	*12 hpi*	*24 hpi*	*48 hpi*
BC_2F_1 TX89D6435 × Euforia	367	367	266	316	179
BC_2F_1 Purdue × Euforia	615	755	597	575	152
BC_2F_1 Purdue × Medalistka	639	352	199	264	139
BC_2F_1 TX89D6435 × Medalistka	602	368	292	312	221
BC_2F_1 TX89D6435× Symetria	338	433	282	272	254
BC_2F_1 Purdue × Symetria	715	524	474	462	427
