# Supplementary material for: Genetic Background-Dependent Expression of Lr34, Lr67 and the Lr46-Associated Candidate Gene Lr46-Glu2 in Wheat (Triticum aestivum L.) Highlights Contrasting Transcriptional Responses Following Puccinia triticina Infection
Source: Int J Mol Sci. 2026 Aug 18;27(16):7391. doi: 10.3390/ijms27167391 (PMC13513563; doi:10.3390/ijms27167391)
Supplement: Supplementary file 1 [file ijms-27-07391-s001.zip › Table S6.pdf]

Table. S6. Expression levels of miR9780 in the analysed winter wheat genotypes before inoculation and at 6-, 12-, 24-, and 48-hours post-inoculation (hpi). Values represent means of biological replicates (including technical replicates).

**Plant material**	**miRNA copy number/µL at:**				
	0 hpi	***6 hpi***	***12 hpi***	***24 hpi***	***48 hpi***
BC_2F_1 TX89D6435 × Euforia	19	15	7	5	11
BC_2F_1 Purdue × Euforia	22	6	10	9	4
BC_2F_1 Purdue × Medalistka	28	5	6	6	5
BC_2F_1 TX89D6435 × Medalistka	5	14	3	6	8
BC_2F_1 TX89D6435× Symetria	35	6	15	4	5
BC_2F_1 Purdue × Symetria	20	5	4	3	11
