# Supplementary material for: Genetic Background-Dependent Expression of Lr34, Lr67 and the Lr46-Associated Candidate Gene Lr46-Glu2 in Wheat (Triticum aestivum L.) Highlights Contrasting Transcriptional Responses Following Puccinia triticina Infection
Source: Int J Mol Sci. 2026 Aug 18;27(16):7391. doi: 10.3390/ijms27167391 (PMC13513563; doi:10.3390/ijms27167391)
Supplement: Supplementary file 1 [file ijms-27-07391-s001.zip › Table S7.pdf]

Table. S7. Pearson correlation analysis between tae-miR5384-3p abundance and *Lr46-Glu2* expression across all BC_2F_1 winter wheat genotypes, donor-derived groups, and individual genotypes. Pearson correlation coefficients were calculated using paired tae-miR5384-3p abundance and *Lr46-Glu2* relative expression values obtained at 0, 6, 12, 24 and 48 hpi. The overall analysis included 30 genotype × time-point observations. Analyses for TX-derived and Purdue-derived materials included 15 observations each, whereas genotype-specific analyses included five time points per genotype. Statistical significance was assessed at $P < 0.05$.

**Dataset**	**n**	**Pearson's r**	**P-value**
All genotypes and time points	30	−0.070	0.713
TX-derived genotypes	15	−0.132	0.640
Purdue-derived genotypes	15	0.310	0.261
TX × Euforia	5	−0.585	0.300
TX × Medalistka	5	−0.586	0.299
TX × Symetria	5	0.223	0.718
Purdue × Euforia	5	0.017	0.979
Purdue × Medalistka	5	−0.307	0.615
Purdue × Symetria	5	0.125	0.841
